# Progress in Ophthalmology

**Published:** 1900-07-28

**Authors:** 


### PROGRESS IN OPHTHALMOLOGY.

In his Erasmus Wilson lectures delivered at the Royal College of Surgeons in February last, Mr. Treacher Collins[1] draws attention to many interesting points in the pathology of eye disease, which shed a new light on certain important clinical facts. Speaking of dermoid growths of the eye, he points out that when there is a congenital coloboma of the lid, there is often a growth of skin on the eyeball filling up the notch. In like manner little isolated patches of skin, spoken of as dermoids, occur most frequently opposite the palpebral aperture—that is, in the region of the eye which would remain longest uncovered by the eyelids in their extension over it during fœtal life. He confirms the suggestion of Ryba that these nodules fail to become mucous membrane owing to their never having been covered over by the proper outgrowth of the eyelids. Transverse calcareous films of the cornea, either as a primary change, or occurring as a secondary change in blind eyes (more particularly in blind glaucomatous eyes), consist of portions of Bowman's membrane which have become fibrous and granular. Under the microscope, when treated with hydrochloric acid, bubbles are given off, proving that calcareous material is present. Mr. Collins attributes their existence to persistent œdema of the cornea. Owing to the pressure of the lids this œdema does not occur except over the exposed portions of the cornea, and hence the upper portion of the cornea is never affected by transverse film. His observations on the origin of Descanet's membrane are important. On its inner surface little hyaline nodules of the same structure as the membrane are found, and the conclusion is that both they and the membrane itself are the secretion or product of the endothelial cells lining the back of the cornea. Under certain abnormal conditions the endothelial covering of the cornea causes a deposit of hyaline material on the iris. In nearly all mammals the sclerotic is entirely fibrous, but there are besides white fibrous tissue a number of yellow elastic fibres. These are more abundant near the optic disc, and probably posterior staphyloma and the concentric streaks in rupture in the choroid are to some extent dependent on this fact. In fishes, reptiles, and birds, and amongst mammals in the ornithorrhynchus and echidna, there are usually cartilaginous plates in the sclerotic. It is, therefore, the less surprising that in rare instances congenitally microphthalmic eyes have plates of cartilage in the sclerotic. Cystoid cicatrices, Mr. Collins points out, are invariably due to the sides of a wound having become lined with epithelium. They almost invariably occur at the iritic angle, and are due to the entanglement of a portion of the iris in the first instance. He believes that in some cases these cystic cicatrices are a medium by which infection can be introduced into the eye, and panophthal uritis set up. He further gives it as his opinion that it is by means of cystoid cicatrices that sympathetic ophthalmitis is started. "Any eye," he says, "with a sub-conjunctival entanglement of the uveal tract, is probably liable, as the result of some conjunctivitis in which infective material enters the sub-epithelial tissue, to become the starting point of sympathetic disease, no matter how long a time may have elapsed from the receipt of the original injury." He attributes the decrease in the frequency of sympathetic ophthalmitis to the fact that surgeons are much more careful in avoiding entanglement of the iris than they were formerly. He alludes to the well-known danger of protruding tags of vitreous as the result of needling operations, and to obviate this he recommends that for discission of a membrane the needle be introduced through the sclerocorneal junction. Seeing that secondary cataracts (opaque membranes) are due in some cases to a new growth from the cells lining the anterior capsule of the lens, he advocates the removal of a piece of the capsule with forceps, as practised by Forster.

M. Alfred Blanchard[2] draws attention to optic atrophy occurring after mumps. A month after the onset of the disease vision rapidly failed, and later paresis of one internal rectus and conjunctival injection supervened. Dr. Blanchard refers the origin of the atrophy to the toxic materials produced by mumps, just as optic neuritis occurs after variola measles and influenza.

Partial substitution of the cornea by means of a flap, or graft of conjunctiva, is strongly recommended by Kuhn[3] in perforating ulcer of the cornea with prolapse of iris, and in all similar cases. The shape of the globe is better maintained, pain is lessened, healing is complete in about a week, and the flap gradually vanishes, leaving only a kind of pannus tenuis.

At the Ophthalmological Society in February, Dr. Arnold Lawson[4] related a case of cicatrix horn growing from the cornea. No other such cases are recorded in ophthalmological literature. The patient was a hydrocephalic idiot, aged eight years. A conical tuberculated excrescence half an inch long, rising from the cornea, and covering about four-fifths of its surface, was seen protruding from the lids. The growth apparently consisted of dried-up granulation tissue from a central staphyloma with prolapsed iris, and was not corneal in its origin.

Protargol in 5 per cent. solution is recommended by Dr. A. Maitland Ramsay[5] in the treatment of gonorrhœal ophthalmia in preference to nitrate of silver. The application must be repeated more frequently than is permissible with the nitrate. Speaking of the cicatricial changes which follow gonorrhœal ophthalmia in some cases, he affirms that it is chronic blennorrhœa, and not follicular conjunctivitis, which prepares the conjunctiva for the growth of the trachoma microbe.

Some further experiments by Guttmann with the local anæsthic holocaine lead to the conclusion that it is preferable to cocaine for squint operations, but not for conical operations, where the increased vascularity of the conjunctiva is a great objection. Peronin,[6] a new local anæsthetic, produces deep and well-marked anæsthesia, but causes so much injection of the conjunctiva and smarting of the eye that it is not suitable for ordinary eye operations.

[1] Lancet, 1900, p. 435. [2] Le Bulletin Med., Dec., 1899. [3] Ophthal. Review, Oct. [4] Lancet, Feb. 3, 1900, p. 311. [5] Edin. Med. Jour., Jan., 1900. [6] Brit. Med. Jour., Oct. 28, 1899, Epitome.

